# Editorial for the special issue on “Prediction, Creativity, and Cultural Evolution in Music Cognition”

**DOI:** 10.5334/joc.399

**Published:** 2024-09-09

**Authors:** Tudor Popescu, Andrea Schiavio, Felix Haiduk

**Affiliations:** 1 Department of General Psychology, University of Padova, Via Venezia, Padova, Italy; 2 Vienna Cognitive Science Hub, University of Vienna, Kolingasse, Vienna, Austria; 3 University of York, York, YO10 5DD, United Kingdom; 4 Behavioral and Cognitive Biology, University of Vienna, Wien, Austria

**Keywords:** Music cognition, Auditory perception, Emotion and cognition, Learning, Social cognition, Speech perception

## Abstract

Music making across cultures arguably involves a blend of innovation and adherence to established norms. This integration allows listeners to recognise a range of innovative, surprising, and functional elements in music, while also associating them to a certain tradition or style. In this light, musical **creativity** may be seen to involve the novel recombination of shared elements and rules, which can in itself give rise to new cultural conventions. Put simply, future *norms* rely on past *knowledge* and present *action*; this holds for music as it does for other cultural domains. A key process permeating this temporal transition, with regards to both music making and music listening, is **prediction**. Recent findings suggest that as we listen to music, our brain is constantly generating predictions based on prior knowledge acquired in a given enculturation context. Those predictions, in turn, can shape our appraisal of the music, in a continual perception-action loop. This dynamic process of predicting and calibrating expectations may enable shared musical realities, that is, sets of norms that are transmitted, with some modification, either vertically between generations of a given musical culture, or horizontally between peers of the same or different cultures. As music transforms through **cultural evolution**, so do the predictive models in our minds and the expectancy they give rise to, influenced by cultural exposure and individual experience. Thus, creativity and prediction are both fundamental and complementary to the transmission of cultural systems, including music, across generations and societies.

For these reasons, ***prediction, creativity and cultural evolution*** were the central themes in a symposium we organised in 2022. The symposium aimed to study their interplay from an interdisciplinary perspective, guided by contemporary theories and methodologies. This special issue compiles research discussed during or inspired by that symposium, concluding with potential directions for the field of music cognition in that spirit.

## 1. Summary of articles

1. Correa examines how cultural and individual differences affect musical expectancy and prediction during multimodal listening. The study focuses on non-metrical, non-tonal “*complex-sound” music*, thereby critically extending the traditional focus of music cognition research. Using grounded theory, post-listening interviews, and self-relevance appraisal, Correa identifies Cross-Modal Musical Expectancies (CMMEs) as key in explaining prediction. CMMEs are influenced by both acoustic signals and the listener’s cultural and personal history, broadening traditional expectancy models by including cross-modal and episodic memories.
2. Homer et al. present a computational theory of knowledge representation through geometric modeling of pitch. Building on Large and Milne’s work, their cognitive architecture models perceptual and cognitive knowledge as emerging from mathematical structures applied to empirical data. Neural assembly dynamics processing perceived input are described using resonance spaces, representing sound fragments as oscillatory functions. Their model explains empirical observations and reassuringly captures harmonic distance, fitting classical methods of assessing inter-chord and inter-key distance. This work forms part of a broader framework (the Information Dynamics of Thinking, IDyOT), which models creativity from learned information, underscoring the cognitive plausibility of resonance spaces for musical creativity.
3. Matthews et al. explore the “pleasurable urge to move to music” (PLUMM) in relation to rhythmic complexity and predictive processing. They propose that PLUMM follows an inverted U-shaped curve, with moderately complex rhythms eliciting the strongest urge due to an optimal balance between predictability and surprise. This is explained by the learning progress (LP) hypothesis, linking pleasure to the brain’s ability to minimise prediction errors. The role of dopamine and norepinephrine in reinforcing memory and learning through rhythmic prediction errors is highlighted. The paper also discusses how dancing enhances social bonding and collective LP by providing a shared predictive framework.
4. Passmore and Savage investigate global musical diversity by examining commonalities and differences in musical cultures. Analysing rhythm, melody, and harmony across a robust global dataset, they identify universal trends and anomalies, influenced by geographical, historical, and social contexts. Significant findings include specific musical elements that challenge existing theories of musical universality, suggesting a nuanced understanding of musical evolution. Their work underscores the balance between shared human experiences and unique cultural expressions.
5. Pham and Fletcher propose a new approach to understanding prehistoric acoustic environments. They argue that traditional studies focusing on material artifacts and sociocultural functions are insufficient. Introducing the concept of “negative space” from art history, they emphasise the depth and three-dimensionality of acoustic environments. They advocate a “spectralist” approach, acknowledging the continuous nature of sound and incorporating both intentional and ambient noises. Their framework broadens archaeoacoustic research, considering the full spectrum of sound in human history, for a holistic understanding of ancient acoustic environments.
6. Singer et al. explore the link between predicting musical event timing and the experience of pleasantness. Using tapping and continuous ratings of valence and arousal, they find a significant positive correlation between temporal predictability and aesthetic valence. This suggests that predictable musical moments are more pleasant. This correlation holds across various musical segments and is validated with a large song database. The study highlights the enhancement of hedonic musical experiences through rhythmic predictability and discusses its social and evolutionary implications for group synchronisation and bonding.
7. Wanke investigates perceptual and cognitive mechanisms in engaging with “sound-based” genres like post-spectralism and electroacoustic music. These genres lack linear construction and common expectations, instead presenting dynamic planes and shapes. Wanke argues that such “geometries in motion” are best understood through morphodynamic theory, a connectionist model of cognition engaging listeners phenomenologically. This theory suggests that sound patterns evoke mental representations reflecting Gestalt and kinaesthetic principles. Through a listening survey, Wanke shows that these genres bridge the acoustic-physical world and symbolic cognition, challenging traditional cognitive models and proposing a new understanding of contemporary art music.

## 2. A note on the variety of methodologies employed

The methodologies employed across the seven papers in this special issue are diverse and often interdisciplinary, reflecting the multifaceted nature of music cognition research. Correa and Wanke both conduct qualitative analyses, the former applying it to examine the cognitive processes underlying musical expectancy and creativity, and the latter calling for the study of sound-based music within a connectionist model of cognition. Pham and Fletcher combine archaeology and acoustics to explore ancient music, borrowing a concept from art history and building a framework tested in a case study. Homer et al. build on work dealing with the representation of knowledge in Artificial Intelligence (AI) systems, and in particular deal with spectral knowledge as emerging from neural oscillations. Matthews et al. integrate psychological and neuroscientific theories to propose a comprehensive model explaining how rhythmic complexity, predictive processing, and the urge to move to music are interconnected. Passmore and Savage employ phylogenetic measures to estimate cultural isolation, and use feature frequency to compute the “unusualness” of songs, whose importance is compared across three different theories of cultural change using Bayesian multi-level regression. Finally, Singer et al. extracted several musical features and computed inter-subject coherence for them, with permutation testing based on phase-randomization. This methodological variety is necessarily wide, covering the broad scope of the special issue’s interest in understanding how prediction, creativity, and cultural evolution intersect in music cognition.

## 3. Conclusion and future directions

The research presented in this special issue highlights the intricate relationship between prediction, creativity, and cultural evolution in music cognition. By examining how cultural and personal factors shape musical expectancy and creativity, these studies offer valuable insights into the mechanisms of cultural transmission and innovation. Future research in any of these fields, or their combination, might continue to explore these themes based on the following approaches:

1. **Cross-Cultural Studies**: Investigating music cognition across different cultures can reveal shared principles and cultural specificities in how music is perceived, created, and transmitted. This can enhance our understanding of the role of cultural context in shaping musical experiences and inform theories of cultural evolution.
2. **Longitudinal Studies**: Examining how musical preferences and predictive models evolve over time within individuals and societies can shed light on the phylogenetic dynamics of cultural transmission and innovation. Longitudinal studies can help identify the factors that drive changes in musical tastes and the adoption of new musical styles and draw direct causal relationships.
3. **Harness new computational technology**: Using recent advances in machine learning, data science, and AI, can facilitate the discovery of patterns and latent relationships of similarity and difference that are hidden in the structures of the world’s music.
4. **Interdisciplinarity**: Integrating insights from neuroscience, psychology, anthropology, and computational modelling is key to provide a comprehensive understanding of music cognition. For instance, combining neuroimaging techniques with computational models like IDyOT can elucidate the neural correlates of creative processes in music. Fulfilling this aspiration requires both theoretical foundations to establish reference points across fields as well as a publishing infrastructure to foster such work.
